# The relationship between bornout, somatic symptoms and work stress among hospital medic staff.

**DOI:** 10.1192/j.eurpsy.2024.1411

**Published:** 2024-08-27

**Authors:** I. A. Török, K. Németh-Rácz, E. Kelemen, E. Szabóné Frank, I. Szendi

**Affiliations:** ^1^Psychiatry Departmentt, Semmelweis Hospital Kiskunhalas, Kiskunhalas; ^2^-, -, -, Hungary

## Abstract

**Introduction:**

The mental health for workers in the healthcare industry have been put through challenges.The first evaluation happened during the first wave of the pandemic, the second one, with grater sample size, have been conducted in Spring 2022.The healthcare system makes it less plausible to release stress adequately. The attitude of repression by the people makes the rise in stress-levels less knowledgeable This time the somatic symptoms makes the stress-levels steady shown. Our goal, to make visible, to categorise and recognise the somatic symptoms and the psychological symptoms, thus predicting the burn-out phase.

**Objectives:**

The attitude of repression by the people makes the rise in stress-levels less knowledgeable This time the somatic symptoms makes the stress-levels steady shown. Our goal, to make visible, to categorise and recognise the somatic symptoms and the psychological symptoms, thus predicting the burn-out phase.

**Methods:**

Methods:

Participants: 497 medic workers

- PPS - Perceived Stress Scale - Type d personality scale -
- Workplace Stress Questionnaire and Symptom List (Hungarian Hypertonia Society)
- Beck Depression Questionnaire (9-item)
- Oldenburg Burn-Out Questionnaire Results: From the questionnaire answers we counted
- WHO Well-being Scale (5-item)

**Results:**

12% of the people reached levels above the significant stress-level and 26% reached the mild-depression level. The burn-out levels have been significantly higher in the region of disappointment. Regarding the results of the somatic symptoms, depression and stress levels it had a leading factor, which was exhaustion.

The most frequent co-occurences of the 20 somatic and psychological symptoms of the Hungarian Hypertension Society Symptom List were also used in this study to refine the analysis. The factor analysis highlited 3 sypmtom clusters out of the 20 symptoms with the following co-occurrences (fatigue, concentration disturbance, headache, feeling of tension, palpitation, dizziness, inner tremor, distressing thoughts, sweating and nausea) The symptoms formed a total of 6 factors, of which 2 were found to be predictive of burnout and depression. The factors of muscle tension, fatigue, lack of concentration, feeling tense showed the strongest correlation with the measured varibles (burnout r=0,447, depression r=0,343, D-scale, negative mood r=0,369, p=0,000 at significance levels.)

**Conclusions:**

The attention for the somatic complaints have a high attention between the workers, it’s part of the work culture to give more and more sacrifices, to hide the psychological effects, and deem them as weaknesses. Regarding the health of the worker it’s necessary to be more informative, to show more bearable physical symptoms to define and prevent the burn-out periods.

**Disclosure of Interest:**

None Declared

